# Ewing Sarcoma Developed at the Site of Previous Mast Cell Proliferation

**DOI:** 10.7759/cureus.50537

**Published:** 2023-12-14

**Authors:** Ridhi Ranchor, Manuel Magalhães, Eugénia Rosendo, André Coelho, Pedro Cardoso

**Affiliations:** 1 Department of Medical Oncology, Centro Hospitalar Universitário de Santo António, Porto, PRT; 2 Department of Pathological Anatomy, Centro Hospitalar Universitário de Santo António, Porto, PRT; 3 Department of Orthopedic Surgery, Centro Hospitalar Universitário de Santo António, Porto, PRT

**Keywords:** ewing sarcoma, mast cell proliferation, mastocytosis, cd117, kit gene mutation, sarcomatous evolution

## Abstract

KIT gene mutations in Ewing sarcomas are rare; however, they are much more frequent in other neoplasms, namely mastocytosis. We describe a case of an adult male with a one-year duration of recurrent episodes of pain, swelling, and redness on the proximal phalanx of the third finger of his right hand. A core biopsy suggested a possible mastocytosis. After four years of recurrent episodes and worsening symptoms, an incisional biopsy revealed an Ewing sarcoma with a KIT gene mutation (M541L, on exon 10). KIT gene mutations with gain-of-function were identified in 2.6% of Ewing sarcomas. In this case, the detection of a KIT mutation in an Ewing sarcoma developed at the site of previous mast cell proliferation raises the hypothesis of a possible sarcomatous evolution of the original lesion. To the best of our knowledge, similar cases are not described in the current literature. This is also the first report describing the KIT M541L mutation (exon 10) in Ewing sarcoma.

## Introduction

Ewing sarcoma is a rare solid tumor, with the majority of cases occurring in bone (15%-20% of cases develop in soft tissues) [1]. It corresponds to the second most common bone neoplasm that mainly occurs in children and young adults, especially in the second decade of life [2]. This neoplasm can affect any bone; however, the axial skeleton, pelvis, and femur are the most commonly affected sites [3].

Although distant metastases are present in 25% of cases (most often in lung, bone, and bone marrow), Ewing sarcoma exhibits a high rate of recurrence (over 90%) if treated without systemic therapy, emphasizing an aggressive pattern of this disease with a high potential to develop micrometastases [4,5].

KIT gene somatic mutations with gain-of-function lead to constitutive activation of the tyrosine kinase receptor, playing a key role in carcinogenesis and sustained tumor growth. The presence of KIT mutations in Ewing sarcomas is rare, but much more frequent in other neoplasms, namely mastocytosis [6-9].

We aimed to describe a case of an adult with an Ewing sarcoma with a KIT mutation developed in a local of a previous mast cell proliferation.

## Case presentation

**Figure 1:**
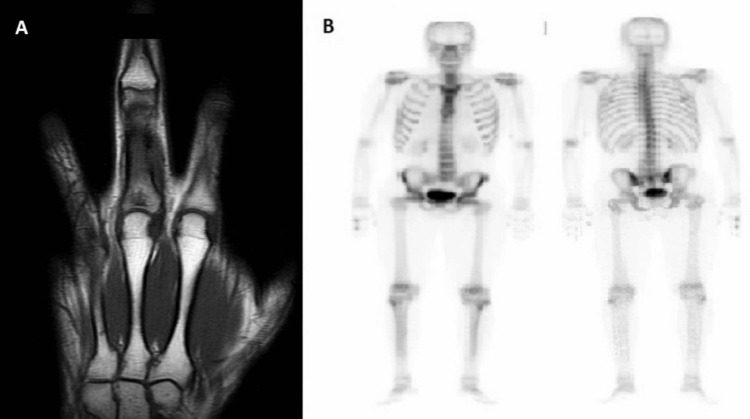
(A) Coronal T1-weighted magnetic resonance imaging of the right hand (2016). (B) Bone scintigraphy (2016).

A male in his early 30s presented in 2016 with pain on the proximal phalanx of the middle finger of the right hand associated with redness and swelling, with a one-year duration. Blood analysis was normal for a complete blood count, renal and liver function, electrolyte balance, C-reactive protein, and tryptase. Magnetic resonance imaging (MRI) of the right hand found an abnormal morphology at the base of the first phalanx of the middle finger and an oval area measuring 7 mm. In addition, there was a focal area of rupture of cortical bone and an increase in adjacent soft tissue volume. There was no onion peel periosteal reaction (Figure 1, Panel A). Bone scintigraphy demonstrated two areas of hyperactivity on the first phalanx of the middle finger of the right hand (Figure 1, Panel B). A core biopsy was performed, and histological examination revealed a few aggregates of small- to intermediate-sized cells, epithelioid to fusiform, with immunoreactivity only for CD117, suggesting a possible mastocytosis. Systemic mastocytosis could not be confirmed by subsequent bone marrow evaluations or by any other organ involvement.

Due to recurrent episodes of pain, swelling, and redness at the same site refractory to antihistamines, topical and oral corticosteroids, and sodium cromoglycate, the case was discussed again at a multidisciplinary team consultation in 2018, and local corticosteroid administration was initiated. However, the patient maintained recurrent attacks without change in frequency or severity. In 2020, the pain worsened, and a rapidly growing swelling appeared at the same location.

**Figure 2:**
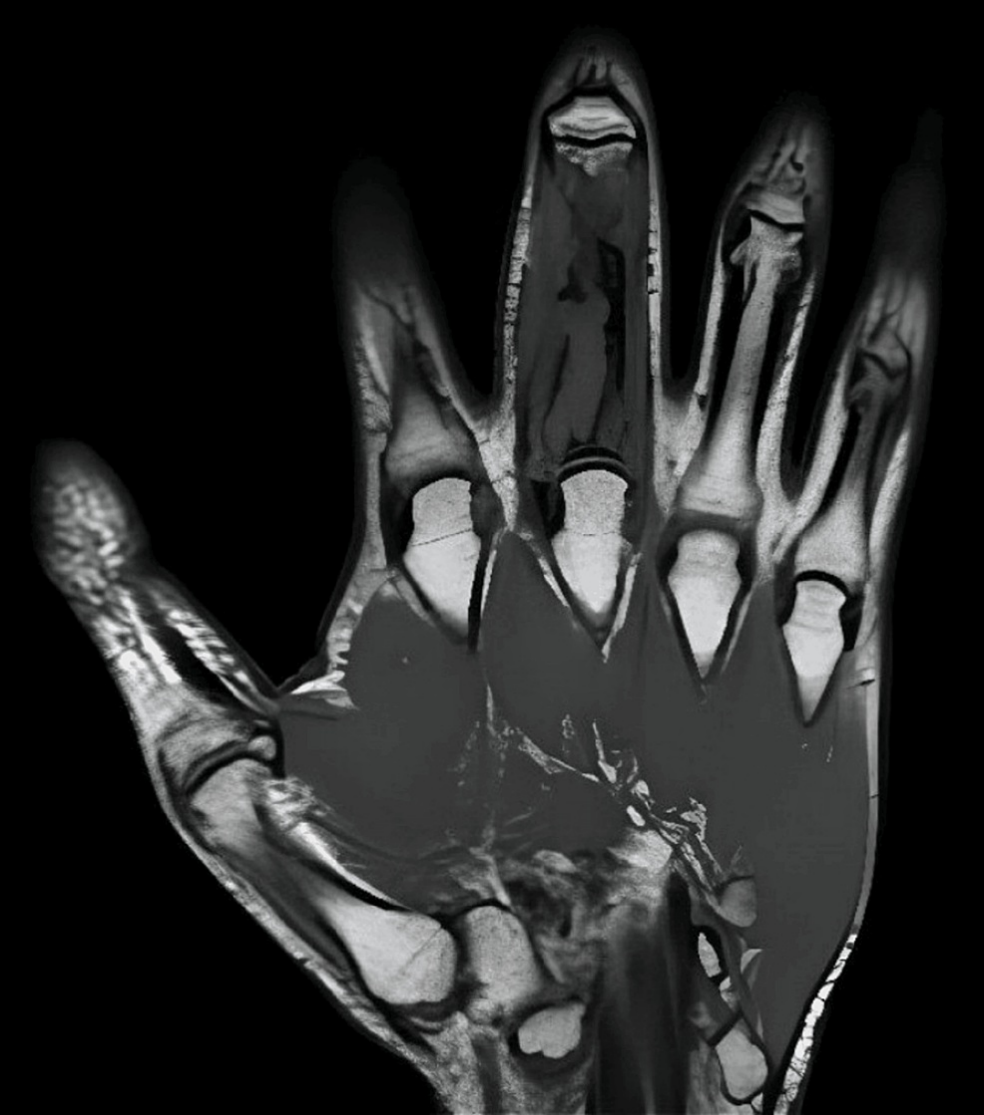
Coronal T1-weighted magnetic resonance imaging of the right hand (2020)

MRI demonstrated a diffuse infiltration of the bone marrow of the entire proximal phalanx of the middle finger of the right hand, with the longest diameter in the axial section of 25 mm. It also showed a complete replacement of the normal pattern of adipose signal and two hyper-uptake areas: the base of the phalanx and its distal third. Areas of cortical bone discontinuity and exuberant soft tissue involvement were also visualized (Figure 2). 18-Fluorodeoxyglucose positron emission tomography/computed tomography (18F-FDG PET/CT) revealed very intense uptake in the proximal phalanx of the middle finger of the right hand, with a maximum standardized uptake value (SUVmax) of 11.3. There was no sign of distant metastasis.

**Figure 3:**
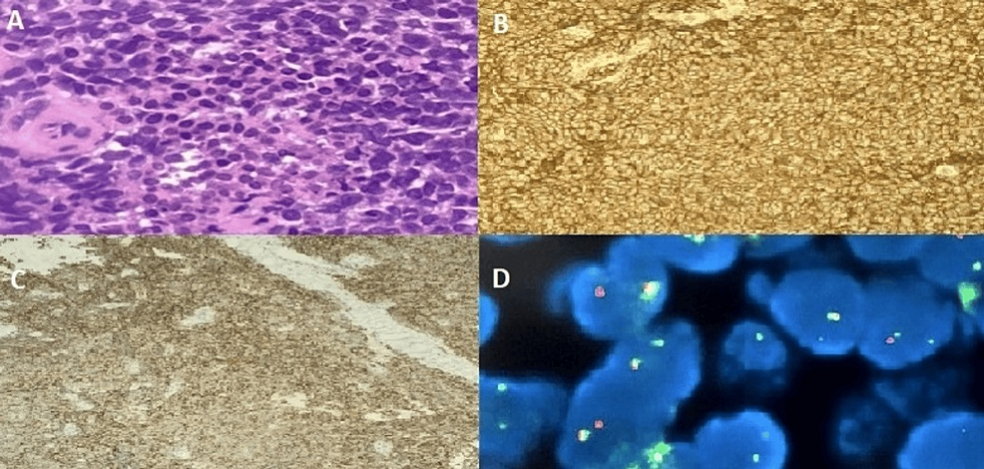
Histological examination of incisional biopsy (A) Hematoxylin and eosin stain (400x). (B) Immunohistochemistry stain for CD99 (12E7, DAKO, Glostrup, Denmark), positive control: esophageal mucosa, platform: Ventana BenchMark Ultra (400x). (C) Immunohistochemistry stain for CD117 (YR145, Cell Marque, Rocklin, CA), positive control: appendix, platform: Ventana BenchMark Ultra (200x). (D) Fluorescence in situ hybridization analysis using Vysis LSI EWSR1 (22q12) Dual Color Break Apart Rearrangement Probe (Abbott Molecular, Abbott Park, IL): nuclei with EWSR1 gene rearrangement exhibit separation of green and orange signals (600x).

According to these findings, the patient was submitted to an incisional biopsy. Histology demonstrated dense cellular proliferation organized in solid nodules of small, round, and monomorphic cells with scanty, clear cytoplasm and a nucleus with regular contours without an apparent nucleolus (Figure 3, Panel A). Immunohistochemistry (IHC) revealed immunoreactivity for CD99, NKX2.2, and CD117 (Figure 3, Panels B and C). A translocation involving the chromosomal region 22q12 (EWSR1 gene) was detected by fluorescence in situ hybridization (FISH) (Figure 3, Panel D). The KIT gene mutation (M541L, on exon 10) was detected by next-generation sequencing (NGS).

**Figure 4:**
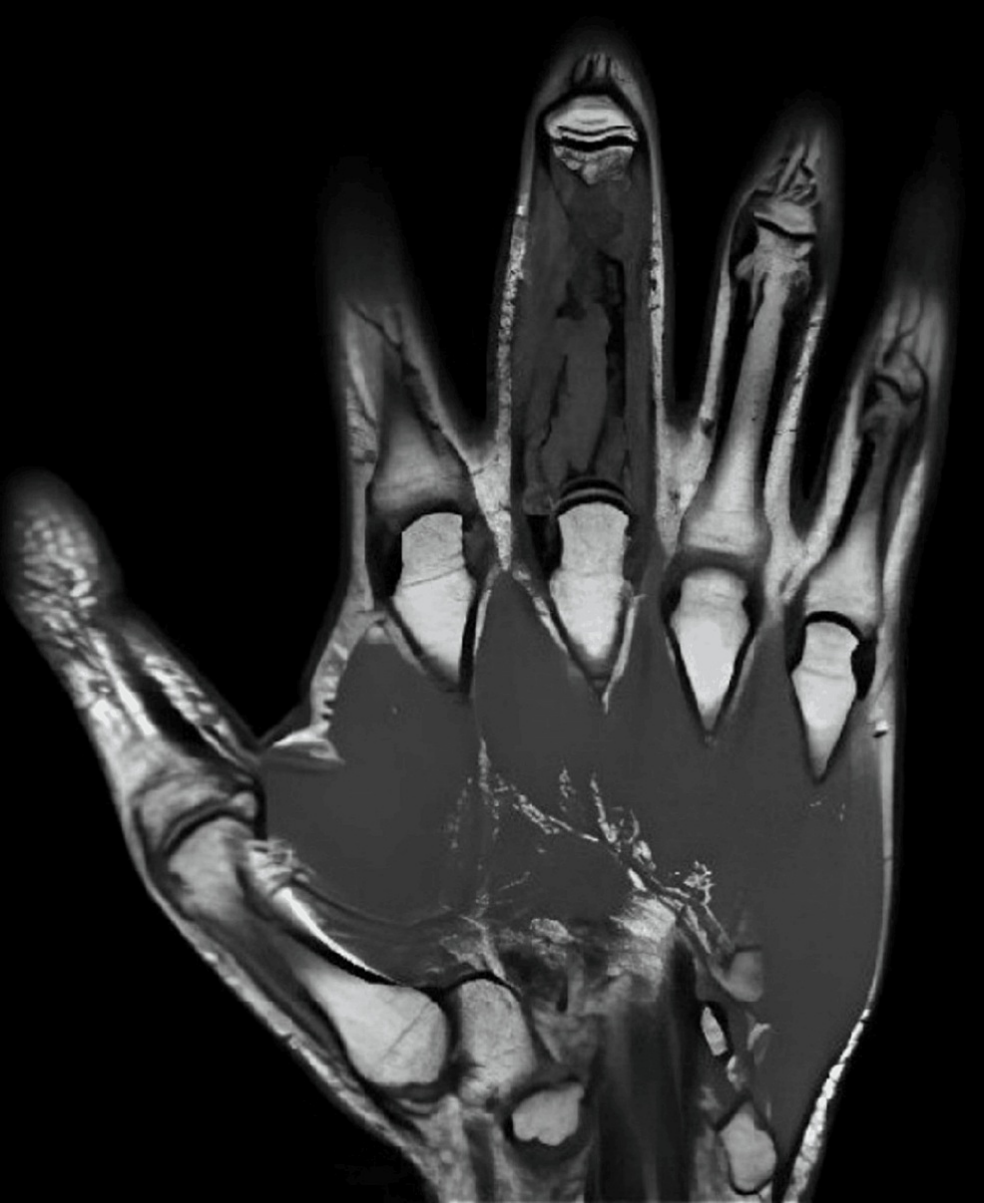
Coronal T1-weighted magnetic resonance imaging of the right hand after neoadjuvant treatment

The patient started neoadjuvant treatment with a combination of alternating chemotherapy every three weeks. This regimen included vincristine, doxorubicin, and cyclophosphamide (VAC; vincristine at a total dose of two mg on day one, doxorubicin 75 mg/m^2^ on days one and two, and cyclophosphamide 1200 mg/m^2^ on day one) alternating with ifosfamide plus etoposide (IE; ifosfamide 1800 mg/m^2^ infused over one hour and etoposide 100 mg/m^2^ infused over two hours, both daily, for five consecutive days). After completing four preoperative cycles with good clinical and analytical tolerance, an MRI was performed. It showed a dimensional regression of the initial lesion, now with the longest diameter in the axial section of 17 mm, consistent with clinical improvement (Figure 4).

Surgery was performed with the amputation of the middle finger of the right hand without intra- or postoperative complications. Histology revealed a ypT1N0 stage, with tumor necrosis superior to 90% and clear surgical margins. The patient restarted systemic treatment, completing a total of eight postoperative cycles.

After two years since the last systemic treatment, the patient has no evidence of recurrent malignant disease. He maintains regular medical visits every three months for clinical history, physical examination, blood analysis, and imagiological reevaluations (with 18F-FDG PET/CT). The patient remains asymptomatic and maintains independence for all daily and labor activities.

## Discussion

The cell of origin of Ewing sarcoma is not established, although several hypotheses are currently considered (possible origin on mesenchymal cell, hematopoietic cell, fibroblast, or neural crest) [10]. Histologically, these neoplasms are characterized by the presence of small, uniform, undifferentiated round cells unlike other types of sarcomas that may exhibit specific differentiation of a lineage [11]. CD99 expression by Ewing sarcoma cells can be considered a marker, although its specificity is not exclusive to this cancer subtype [3,12]. The genetic hallmark is the translocation involving the EWSR1 gene. In 85%-90% of cases, the chromosomal translocation observed is between chromosomes 11 and 22, t(11, 22)(q24q12), originating from a chimeric fusion gene (EWSR1-FLT1) [13,14]. This encodes an aberrant transcription factor that leads to the proliferation and differentiation of Ewing sarcoma cells. In this case, the characteristics of cells (small, round, and monomorphic, with scantly clear cytoplasm and nucleus with regular contours, without apparent nucleolus), along with immunoreactivity for CD99 and identification of translocation involving the chromosomal region 22q12 (EWSR1 gene) by FISH, support the diagnosis of Ewing sarcoma.

CD117, a transmembrane receptor with tyrosine kinase activity, is encoded by the proto-oncogene c-KIT and is expressed by the majority of hematopoietic cells [15]. With the maturation of hematopoietic cells, only mast cells retain this receptor [16]. Other cells, such as melanocytes, germ cells, some epithelial cells, and Cajal cells of the gastrointestinal tract, also express CD117. By binding to its ligand, stem cell factor (SCF), the transmembrane receptor with tyrosine kinase activity induces cell survival, proliferation, and maturation [15]. Somatic mutations with the gain-of-function of the KIT gene lead to constitutive activation of the receptor, playing a pivotal role in tumorigenesis [6]. CD117 expression is not a specific marker as it is expressed by several types of neoplasms, such as mastocytosis [15]. Despite its low specificity, the expression of CD117 and KIT gene mutations are markers of mastocytosis [7].

Despite the variable expression of CD117 (referred to in up to 65% of cases), KIT gene mutations with gain-of-function were identified only in 2.6% of cases of Ewing sarcoma [8,9]. The case described here illustrates an Ewing sarcoma with a very uncommon presentation that develops secondarily at the site of previous mast cell proliferation after four years of evolution. In common, they share not only the localization but also the expression of CD117. The diagnosis of Ewing sarcoma as an initial lesion is excluded by different histological characteristics and the sudden expansion of the lesion after four years of progressive local inflammatory symptoms. The development of Ewing sarcoma in patients with pre-existing lesions is exceptional. It is described in the literature, mainly in children and young people, in the post-therapeutic context (chemotherapy or radiotherapy) of hematological diseases (non-Hodgkin's lymphomas, diffuse large B-cell lymphoma, and T-cell leukemia). One of the mechanisms involved is the alteration of innate and acquired immunity that fails to detect and eliminate new mutated cells carrying the characteristic translocation of Ewing sarcoma, allowing the initiation of a secondary sarcomatous lesion [17-19].

Multiple KIT gene-activating mutations have been described in mast cell neoplasms. These mutations are frequently found on exons 11 and 17, with the D816V mutation on exon 17 occurring in the majority of cases. Mutations involving exon 10, which involves the transmembrane domain, are rare and sporadically reported in adult mastocytosis. The M541L mutation on exon 10, present in the case reported here, was described in the literature in mast cell neoplasms for the first time in 2010 [20]. As far as we know, there are no previous reports describing the KIT M541L mutation involving specifically exon 10 in Ewing sarcoma.

## Conclusions

Despite the low specificity of CD117, its expression and KIT gene mutations are markers of mastocytosis. Expression of CD117 is variable in Ewing sarcoma, but KIT gene mutations with gain-of-function were identified only in 2.6% of these patients. In this case, the detection of a KIT gene mutation in Ewing sarcoma developed secondarily at the site of previous mast cell proliferation raises the hypothesis of a possible sarcomatous evolution of the original lesion. To the best of our knowledge, this is the first report describing the KIT M541L mutation (exon 10) in Ewing sarcoma.

## References

[1] Akther MJ, Kumar LU, Shaikh SU (2016). Extraskeletal Ewing’s sarcoma of the little finger, a rare case. Glob Surg.

[2] Karski EE, Matthay KK, Neuhaus JM, Goldsby RE, Dubois SG (2013). Characteristics and outcomes of patients with Ewing sarcoma over 40 years of age at diagnosis. Cancer Epidemiol.

[3] Riggi N, Suvà ML, Stamenkovic I (2021). Ewing's sarcoma. N Engl J Med.

[4] Zöllner SK, Amatruda JF, Bauer S (2021). Ewing sarcoma-diagnosis, treatment, clinical challenges and future perspectives. J Clin Med.

[5] Bacci G, Ferrari S, Longhi A (2003). Therapy and survival after recurrence of Ewing's tumors: the Rizzoli experience in 195 patients treated with adjuvant and neoadjuvant chemotherapy from 1979 to 1997. Ann Oncol.

[6] Rihuete C, Acín-Gándara D, Pereira F, Tardío JC (2019). Ewing's sarcoma: differential diagnosis of gastrointestinal stromal tumors (GIST). Cir Esp (Engl Ed).

[7] Horny HP, Sotlar K, Valent P (2014). Mastocytosis: immunophenotypical features of the transformed mast cells are unique among hematopoietic cells. Immunol Allergy Clin North Am.

[8] Ahmed A, Gilbert-Barness E, Lacson A (2004). Expression of c-kit in Ewing family of tumors: a comparison of different immunohistochemical protocols. Pediatr Dev Pathol.

[9] Do I, Araujo ES, Kalil RK, Bacchini P, Bertoni F, Unni KK, Park YK (2007). Protein expression of KIT and gene mutation of c-kit and PDGFRs in Ewing sarcomas. Pathol Res Pract.

[10] Rani PSV, Shyamala K, Girish HC, Murgod S (2015). Pathogenesis of Ewing sarcoma: a review. J Adv Clin Res Insights.

[11] Tu J, Huo Z, Gingold J, Zhao R, Shen J, Lee DF (2017). The histogenesis of Ewing sarcoma. Cancer Rep Rev.

[12] Machado I, López-Guerrero JA, Llombart-Bosch A (2014). Biomarkers in the Ewing sarcoma family of tumors. Curr Biomark Find.

[13] Kelleher FC, Thomas DM (2012). Molecular pathogenesis and targeted therapeutics in Ewing sarcoma/primitive neuroectodermal tumours. Clin Sarcoma Res.

[14] Rizk VT, Walko CM, Brohl AS (2019). Precision medicine approaches for the management of Ewing sarcoma: current perspectives. Pharmgenomics Pers Med.

[15] Miettinen M, Lasota J (2005). KIT (CD117): a review on expression in normal and neoplastic tissues, and mutations and their clinicopathologic correlation. Appl Immunohistochem Mol Morphol.

[16] Dirnhofer S, Zimpfer A, Went P (2006). [The diagnostic and predictive role of kit (CD117)]. Ther Umsch.

[17] Wolpert F, Grotzer MA, Niggli F, Zimmermann D, Rushing E, Bode-Lesniewska B (2016). Ewing's sarcoma as a second malignancy in long-term survivors of childhood hematologic malignancies. Sarcoma.

[18] Khandwala K, Hilal K, Fadoo Z, Minhas K (2018). Metachronous renal Ewing sarcoma/primitive neuroectodermal tumour in a survivor of Burkitt lymphoma. BMJ Case Rep.

[19] Hiramoto N, Kobayashi Y, Nomoto J (2013). Ewing sarcoma arising after treatment of diffuse large B-cell lymphoma. Jpn J Clin Oncol.

[20] Rocha J, Luz Duarte M, Marques H, Torres F, Tavares P, Silva A, Brito C (2010). Association of adult mastocytosis with M541L in the transmembrane domain of KIT. J Eur Acad Dermatol Venereol.

